# Forensic Engineering of Advanced Polymeric Materials—Part V: Prediction Studies of Aliphatic–Aromatic Copolyester and Polylactide Commercial Blends in View of Potential Applications as Compostable Cosmetic Packages

**DOI:** 10.3390/polym9070257

**Published:** 2017-06-29

**Authors:** Wanda Sikorska, Joanna Rydz, Katarzyna Wolna-Stypka, Marta Musioł, Grażyna Adamus, Iwona Kwiecień, Henryk Janeczek, Khadar Duale, Marek Kowalczuk

**Affiliations:** 1 Centre of Polymer and Carbon Materials, Polish Academy of Sciences, 34, M. Curie-Sklodowska St., 41-819 Zabrze, Poland; jrydz@cmpw-pan.edu.pl (J.R.); katarzyna.wolna.stypka@gmail.com (K.W.-S.); mmusiol@cmpw-pan.edu.pl (M.M.); gadamus@cmpw-pan.edu.pl (G.A.); ikwiecien@cmpw-pan.edu.pl (I.K.); henryk.janeczek@cmpw-pan.edu.pl (H.J.); 2 School of Biology, Chemistry and Forensic Science, Faculty of Science and Engineering, University of Wolverhampton, Wolverhampton WV1 1SB, UK; K.Duale@wlv.ac.uk

**Keywords:** biodegradable polyesters, cosmetic packages, organic recycling

## Abstract

The main aim of the present study was to determine the behavior of the specimens from Ecovio, in the form of dumbbell-shaped samples and films, during degradation in selected cosmetic ingredients such as water and paraffin. The (bio)degradation test of the prototype cosmetic package (sachet) made from a PBAT (poly[(1,4-butylene adipate)–*co*–(1,4-butylene terephthalate)]) and PLA (polylactide) blend was investigated under industrial composting conditions, and compared with the sample behavior during incubation in cosmetic media at 70 °C. During the degradation tests, the changes of the samples were evaluated using optical microscopy, ^1^H NMR (proton nuclear magnetic resonance) and GPC (gel permeation chromatography) techniques. The structures of the degradation products were investigated using ESI-MS^n^ (mass spectrometry with electrospray ionization on positive and negative ions) analysis. The thermal properties of selected materials were determined by DSC (differential scanning calorimetry) and TGA (thermogravimetric analysis) analysis. It was concluded that the PBAT and PLA blend studied had a good stability during aging in cosmetic media, and could be recommended for long-shelf-life compostable packaging of cosmetics, especially with oily ingredients.

## 1. Introduction

The replacement of the conventional polymer packing materials by compostable materials has attracted much attention with regard to environmental protection. Currently used cosmetic packaging, made mostly from conventional plastics, have several advantages such as lightness, mechanical strength, and a lack of interaction with their cosmetic ingredients. However, their resistances to biological agents make them a burden to the environment after their periods of use, creating problems associated with the disposal of packaging waste. Moreover, the majority of cosmetic packages contain difficult-to-remove, oily substances (e.g., creams, lotions, oils) that can prevent the material from recycling. This implies a search for new materials that may be used as packaging for cosmetics, and that would not create a threat to the environment. Alternatives to cosmetic packaging from conventional plastics may be the use of biodegradable polyesters [1]. Conversely, we had originally reported that selected cosmetic stimulants, including paraffin, cause morphological and structural transformations in PLA films, which results in damaging the material during degradation. This finding affects the application of PLA as a packaging material in long-shelf-life applications [2,3]. It was also originally reported that blends of PLA with poly[(R,S)-3-hydroxybutyrate] are (bio)degradable materials with proved resistant to degradation in cosmetic simulants [4].

Associations between polymeric materials’ structures, properties and behaviors before, during and after practical applications can be evaluated by the use of the methodology developed by FEAPM (Forensic Engineering of Advanced Polymeric Materials). Optimization and characterization of the biodegradable polymers’ properties are very important for their production, usage and utilization. The connecting of all these elements in the FEAPM methodology constitutes the novelty of this approach. This should help to project new biodegradable polymeric materials, avoiding the product defects generated during production and usage [5,6,7,8].

The compostable aliphatic–aromatic copolyester PBAT was investigated with regard to its application as an improving additive to blends with aliphatic polyesters [9]. PBAT exhibits good mechanical and thermal properties when the content of terephthalic acid is greater than 35 mol %. However, when its content increases, a significant decrease in the rate of biodegradation is observed [10,11]. Comprehensive studies on the PBAT molecular structure were conducted via the analysis of the partially degraded copolymer using proton and carbon nuclear magnetic resonance (^1^H NMR and ^13^C NMR) and the hyphenated techniques of liquid chromatography and mass spectrometry with electrospray ionization on positive and negative ions (LC-ESI-MS^n^). The dyad sequence analysis gave the possibility to determine the molar composition of the copolyester studied. Furthermore, the LC-ESI-MS^n^ enabled the detailed characterization of the chemical structure of the terminal groups of the PBAT copolymer, and the identification of the cyclic oligomers, which were not observed using NMR techniques [12,13].

In a study on PBAT degradation in sandy soil, it was also demonstrated that retention of aromatic chain fragments in the low molar mass fraction of the incubated samples takes place. The results of the phytotoxicity studies show that PBAT degradation products are safe for the tested plants [13]. Until now, only few studies published report the investigation of the Ecovio materials, and these were mostly focused on the mechanical behavior of the composition with different fillers (silica nanoparticles and wood fibers) [14,15,16]. The presence of PBAT in blends reduced the stiffness of PLA and increased the flexibility of the materials obtained [11,17]. The Tachibana group presented the utilization of PLA and PBAT compositions with modified starch as an agricultural mulch sheet for mandarin oranges [18]. The aging test under stressed conditions for commercial PLA bottles, empty and filled, with a standard solution of pH 2 was also performed [1]. However, to the best of our knowledge, the study on the Ecovio materials for cosmetic applications has not yet been published. 

Herein, we report the behavior of dumbbell-shaped samples processed from commercial PLA and PBAT blends (produced under the trademark Ecovio) in contact with selected cosmetic ingredients such as water and paraffin during a laboratory aging test at 70 °C. As mentioned above, in the case of cosmetic products, it is important to determine whether and how the packaging materials will interact with cosmetic ingredients. Such prediction studies of commercial blends were undertaken in view of their potential applications as compostable packages for cosmetics. The (bio)degradation of the selected dumbbell-shaped samples and sachets (made from the same material) were investigated under industrial composting conditions in a Biodegma system at the Station of Mechanical-Biological Waste Treatment in Zabrze. During the degradation tests, macro- and microscopic surface changes of the samples were evaluated, as well as the changes of the blends’ compositions, and the molar masses were determined with the use of ^1^H NMR and GPC techniques. The structure of the degradation products was investigated by ESI-MS^n^. The thermal properties of selected materials were determined by DSC and TGA. Additionally, the proposed approach will be directed toward 3D prototyping of cosmetic compostable packages, and will be presented in the next contribution in this series of publications.

## 2. Materials and Methods

### 2.1. Materials

Ecovio F Mulch C2311, BASF Company (Florham Park, NJ, USA)—a commercial blend of PBAT (containing 47 mol % of aromatic segments) with 12 mol % of PLA, as revealed by NMR analysis—was used. In the study, two types of the specimens were used, one in the form of dumbbell-shaped samples (prepared according to norm [19] with thicknesses of 1.5 mm) marked as EV, and the other in the form of film with thicknesses of 80 μm, as a prototype of the final packaging product (labeled with the short name “sachet”). The thermal properties of plain samples (data from the DSC heating run) are presented in Table 1.

The dumbbell-shaped samples were formed in the center using a micro-extruder Minilab Thermo-Haake (Thermo Fisher Scientific, Waltham, MA, USA) equipped with co-rotating two-cone-shaped screws connected with the MiniJet piston injection molding system (Thermo-Haake).

The film of the PBAT and PLA blend was processed by Bioerg S.A. Company (Dabrowa Gornicza, Poland) on the standard technological line (LUIGI BANDERA SPA, used for the production of the conventional film by extrusion blow molding; see graphical abstract). The line possessed a gravimetric feeding system of raw materials with the automatic system of the speed control and a sufficiently long and temperature-stable plasticizing system. The obtained film was used for the pilot production of a prototype of the final packaging product. The obtained ribbon with a width of 160 mm and a thickness of 80 μm was characterized by mechanical and welding tests (Table 2).

The demineralized water (used as the reference) and liquid paraffin without further purification (99.98%; water content: 0.016% by the Karl Fischer method; from Pharmaceutical Laboratory COEL, Cracow, Poland) were used as cosmetic ingredients in the abiotic degradation experiments. 

### 2.2. Environments

#### 2.2.1. Abiotic Degradation under Laboratory Conditions

The dumbbell-shaped samples (half part) were incubated in demineralized water and paraffin at 70 °C over a period of 52 weeks, according to the ISO norm [20]. The samples were inserted into 30 mL screw-capped vials, each containing 25 mL of the medium, and placed in a laboratory oven at 70 °C. The samples incubated were run in triplicate. After 6, 8, 12, 26 and 52 weeks, the samples were removed from the vials. The samples incubated in water were cleaned by washing in distilled water, draining on blotting paper and then drying to a constant mass under vacuum at a temperature of 25 °C. The samples incubated in paraffin were cleaned by draining on blotting paper, according to [2].

#### 2.2.2. (Bio)degradation under Industrial Composting Conditions

**Figure 1:**
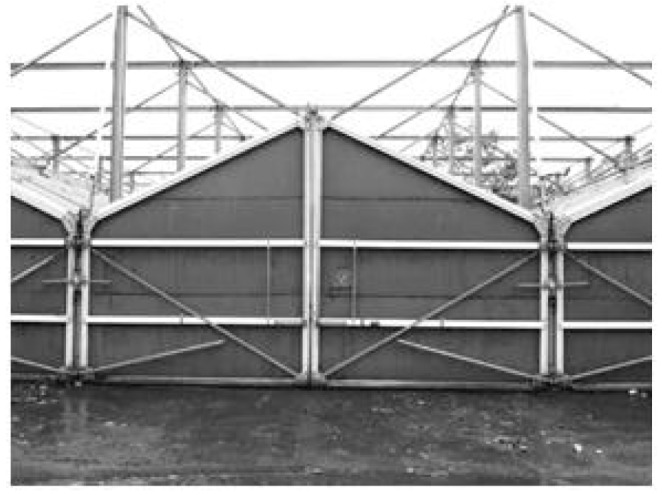
Biodegma system at the Station of Mechanical-Biological Waste Processing in Zabrze.

The study of organic recycling was carried out at the Station of Mechanical-Biological Waste Processing in Zabrze, in the Biodegma closed system with aeration (Figure 1).

The system consists of municipal solid waste, sewage sludge and organic waste. Two types of specimens were used for the composting tests, one in the form of dumbbell-shaped samples, and the second as a pieces of sachets with the dimensions: 4 cm × 3.5 cm. Both types of samples were placed in a testing cage as previously described in [21]. The cages were then located to a roofed tunnel (one module of the Biodegma system; Figure 1) at a depth of 0.5 m below the compost surface. The test of the industrial composting process was carried out in triplicate within 6 weeks, at an average temperature of 61 °C (±5 °C).

### 2.3. Measurements

#### 2.3.1. Imaging of Sample Surfaces

Macroscopic changes to the surface of the materials tested were visualized using a digital camera (Olympus E-410, Tokyo, Japan), while microscopic changes were analyzed using an optical microscope. Analyses were performed using a Zeiss polarizing microscope (Opton-Axioplan, Oberkochen, Germany) equipped with a digital camera (Nikon Coolpix 4500 color, Tokyo, Japan). Pictures were taken at a magnification of 100×.

#### 2.3.2. Gel Permeation Chromatography (GPC) Analysis

The GPC experiments for the samples studied were conducted in a chloroform solution at 35 °C and at a flow rate of 1 mL/min using a Viscotek VE 1122 (Malvern, Worcestershire, UK) pump with two Mixed C PLgel styragel columns (Agilent, Santa Clara, CA, USA) in series and a Shodex SE 61 RI detector (Showa Denko, Munich, Germany). A volume of 10 μL of a chloroform sample solution (concentration 0.5% m/V) was injected into the system. The instrument was calibrated using polystyrene standards with a low dispersity.

#### 2.3.3. Nuclear Magnetic Resonance ^1^H (^1^H NMR) Spectroscopy

^1^H NMR analyses were carried out using a Bruker-Advance spectrometer operating at 600 MHz with Bruker TOPSPIN 2.0 software (Bruker, Billerica, MA, USA). CDCl_3_ was used as the solvent, and tetramethylsilane (TMS) was used as the internal standard. Each spectrum was obtained with 64 scans, a 11 μs pulse width, and a 2.66 s acquisition time. The PBAT and PLA blend composition was determined based on integration of the signal of the methine group of the PLA component (at δ = 5.20 ppm) and the signals of methylene groups in the aromatic and aliphatic dyads presented in the ^1^H NMR spectrum in the region between δ = 4.0–4.5 ppm, i.e., signals 1, 1′, 6, and 6′ ascribed to the respective structures of PBAT (see Figure 5 in Results and Disccusion). The composition of the PBAT component (mol % of aromatic and aliphatic units) was calculated based on the intensities of the signals of the methylene groups in the aromatic and aliphatic dyads (in the region between δ = 4.0–4.5 ppm; see Figure 5) according to [13].

#### 2.3.4. Differential Scanning Calorimetry (DSC) Analysis

DSC measurements were taken with a TA DSC 2010 apparatus (TA Instruments, New Castle, DE, USA) at heating/cooling rates of 10 °C/min. The first calorimetric trace (I-scan, heating run), in which the thermal history was suppressed, was acquired from −70 to 200 °C, and the second calorimetric trace (II-scan, cooling run) was acquired from 200 to −70 °C from melt. All of the experiments were performed under a nitrogen atmosphere (flow of 50 mL/min). The instrument was calibrated with indium standards. The melting endotherm peak temperature maximum was used as the melting temperature (*T_m_*). The glass transition temperature (*T_g_*) was determined as the midpoint of the heat capacity change according to the ASTM E 1356-08 standard [22].

#### 2.3.5. Thermogravimetric Analysis (TGA)

TGA was performed with a TGA/DSC1 Mettler-Toledo thermal analyzer (Columbus, OH, USA) at a heating rate of 10 °C/min in a stream of nitrogen (60 mL/min) from 25 to 600 °C. The obtained TGA data were analyzed with the Mettler-Toledo Star System SW 9.30 (Columbus, OH, USA).

#### 2.3.6. Electrospray Ionization Mass Spectrometry (ESI-MS) Analysis

ESI-MS analysis was performed using a Finnigan LCQ ion trap mass spectrometer (Thermo Fisher Scientific Inc., San Jose, CA, USA). The degradation mediums were dissolved in chloroform/methanol (1:1 *v/v*), and the solutions were introduced to the ESI source by continuous infusion using the instrument syringe pump at a rate of 10 µL/min. The ESI source of the LCQ was operating at 4.5 kV and the capillary heater was set to 200 °C. Nitrogen was used as the nebulizing gas. The analyses were performed in the positive- and negative-ion modes.

## 3. Results and Discussion

Our previous studies revealed that PLA is not necessarily the best candidate for long-shelf-life compostable packaging of cosmetics, especially these with oily ingredients [2,3]. In order to improve the application properties, in the present study, the blend of PLA with the biodegradable, aliphatic–aromatic copolyester PBAT was selected as it should have been more resistant to hydrolytic degradation. The commercially available PBAT and PLA blend was used. The comparative studies of (bio)degradation processes of dumbbell-shaped samples and sachets made from the PBAT and PLA blend were conducted under industrial composting conditions in the Biodegma system. The aging test under laboratory conditions for the abiotic degradation of those samples in selected cosmetic media (water and paraffin) at 70 °C was also conducted. The progress of both degradation experiments was estimated by macro- and microscopic observations of the polymer surfaces, as well as by the molar mass and composition changes of the samples. The structure of the water soluble degradation products was determined at molecular level. The thermal properties of selected materials were also analyzed. The results of the degradation experiments should have verified our hypothesis and given an answer as to whether the PBAT and PLA blend could be a good candidate for cosmetic packaging.

### 3.1. Abiotic Degradation under Laboratory Conditions

**Figure 2:**
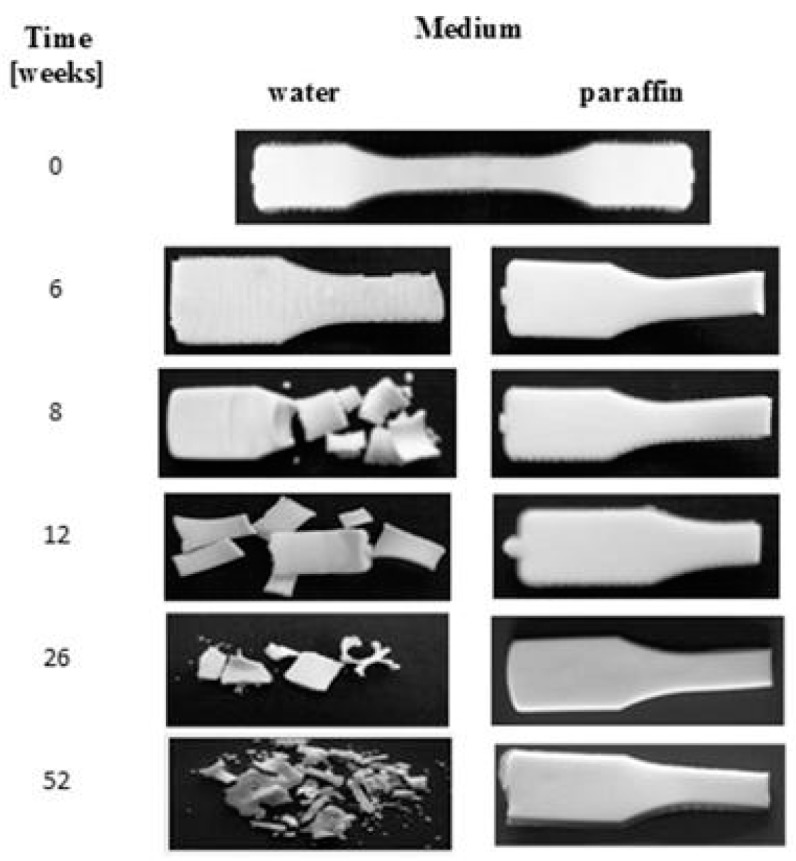
Macroscopic changes of EV samples after specified times of incubation in water and paraffin at 70 °C.

Macroscopic changes for the EV samples after a specified time of incubation in water and paraffin are shown in Figure 2. Before the degradation test, the plain sample was smooth, milky, and slightly shiny. During the incubation in both of the media, no changes in the samples’ surfaces after up to 6 weeks of incubation were noticed. In the 8th week of degradation for the sample in water, cracking of the sample’s surface was noticed, and after 52 weeks, breaking in the sample’s structure, leading to its disintegration and a mass loss of 64%, was observed. In contrast, for the sample degraded in paraffin, neither disintegration nor mass loss during the 52 weeks of incubation was observed.

**Figure 3:**
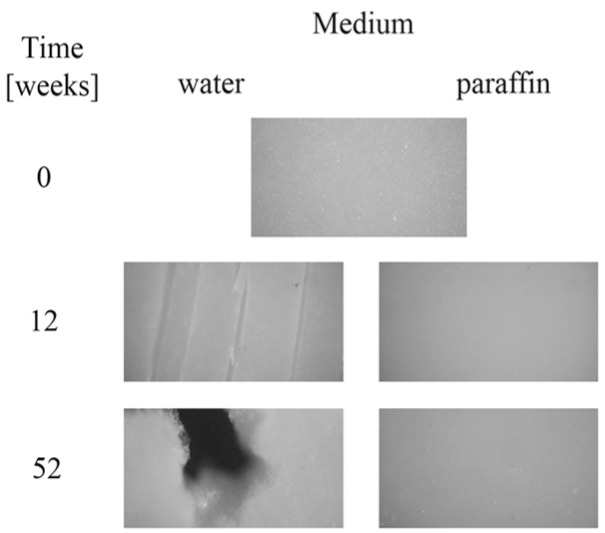
Microscopic changes of EV samples after specified times of incubation in water and paraffin at 70 °C.

Moreover, after longer incubation periods, the samples became more turbid. The observed changes in the transparency could have been caused by molecular reorganization of the samples, or an increase in irregularity in their amorphous phase [23,24]. Microscopic changes of EV samples after 12 and 52 weeks of incubation in water and paraffin are shown in Figure 3. The surface morphology of the plain sample observed under the optical microscope (magnification 100×) revealed a smooth and regular surface. A lack of microscopic changes for samples degraded in paraffin was confirmed; the surface remained smooth and homogeneous. In contrast, on the surface of samples incubated in water, many cracks, cabinet slots or breakthroughs were formed during the early incubation period, and the sample flaked off layer-by-layer. After 52 weeks of the process, holes formed, which led to a partial disintegration of the sample in water.

**Figure 4:**
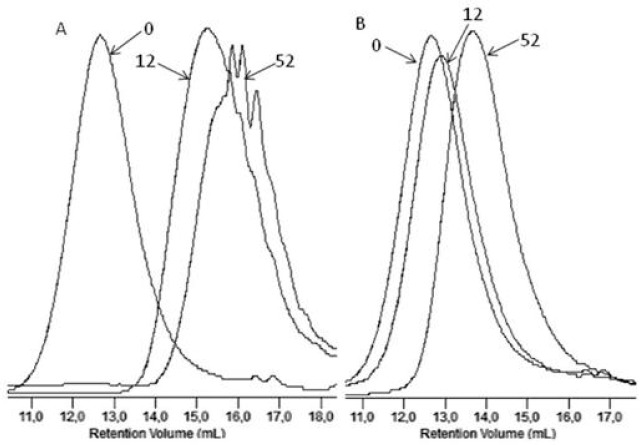
The GPC elugrams of the EV samples before (0) and after 12 and 52 weeks of incubation in water (**A**) and paraffin (**B**).

Figure 4 shows, as an example, the GPC elugrams illustrating the changes in the molar masses of the EV samples before and after specified times of incubation in water and paraffin. A shift of the elution curves towards lower values of the molar masses for samples incubated in water was found, which confirmed that the hydrolysis process in the tested environments occurred, while for samples incubated in paraffin, the noticed reduction of the molar masses had rather minor values that did not lead to a loss of coherence of the material studied (see Figure 4). Additionally, from a comparison of this result with the result obtained in case of PLA, for which the loss of molar mass reached 99% in the 36th incubation weeks (in paraffin), it was noticed that the presence of PBAT improved the stability of the PBAT and PLA blend toward paraffin [2].

**Figure 5:**
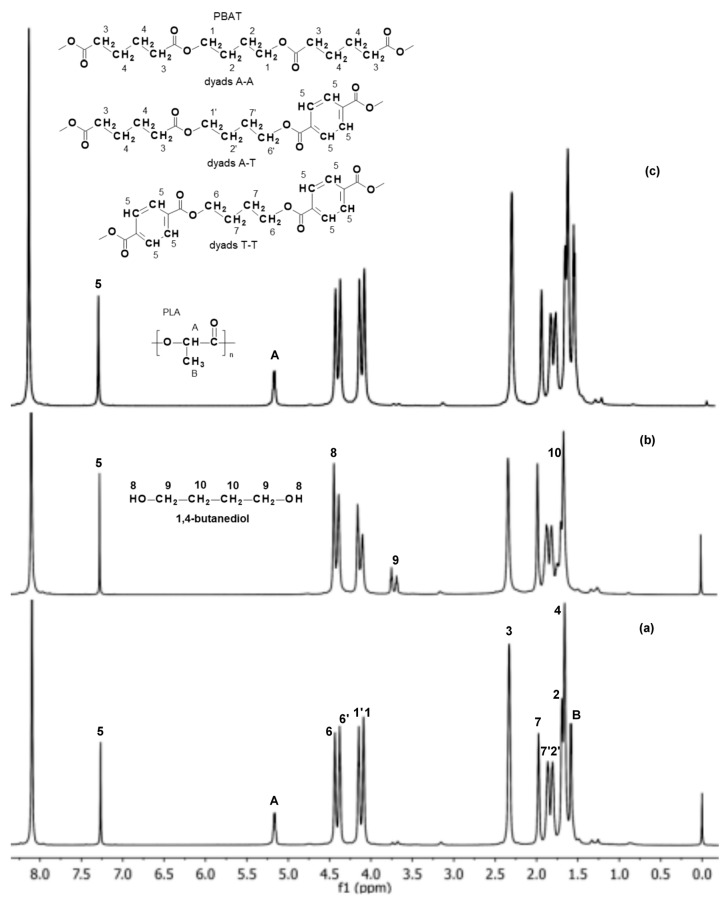
^1^H NMR spectra (extension in the range of 1.0–8.0 ppm) of the EV samples before (**a**) and after 12 weeks of incubation in water (**b**) and paraffin (**c**).

Based on the ^1^H NMR spectra (Figure 5), the molar composition of the PBAT and PLA polymer blend, as well as the mol % of the aromatic and aliphatic comonomer units in the PBAT component were determined, before and after 12 weeks of incubation in water and paraffin. The ^1^H NMR spectrum of the EV sample after 12 weeks of incubation in water showed only the signals 1–7 and 1′, 2′, 6′ and 7′, corresponding to protons of the PBAT blend component (Figure 5b). An appearance of signals characteristic for its degradation product, i.e., 1,4-butanediol (hydroxy group HO–CH_2_– at δ = 4.5 ppm, signal 8; and the methylene group at δ = 3.7 ppm, signal 9), was also noticed. The signals corresponding to protons of PLA were not observed.

Moreover, the intensities of the PBAT component signals, especially those related to the methylene groups in the aromatic and aliphatic dyads A-A, A-T and T-T (signals 1, 1′, 6 and 6′ in the region δ = 4.0–4.5 ppm; Figure 5), were changed during degradation in water. In consequence, the calculated number of aromatic units in the PBAT before and after 12 weeks of degradation was also changed from 47 to 57 mol %, which indicated an increase in the share of aromatic units of the PBAT component of the blend studied. Thus, the composition change with the simultaneous appearance of degradation products from the PBAT may have suggested that hydrolysis of the aliphatic units of the PBAT was privileged. However, in the spectrum of the EV sample after 12 weeks of aging in paraffin, no significant changes were indicated (Figure 5c). It was previously demonstrated that the degradation process of PLA in paraffin occurs as a result of the hydrolysis initiated by a small amount of residual water present in the degradation medium. The hydrophobic characteristic of paraffin suppresses the migration of PLA degradation products, and generates an autocatalytic effect in the polymer matrix [2,25,26]. However, the more hydrophobic PBAT component predominant in the blend caused nearly no significant changes for the samples aging in paraffin. The NMR analysis confirmed the macroscopic observation and the GPC results.

**Figure 6:**
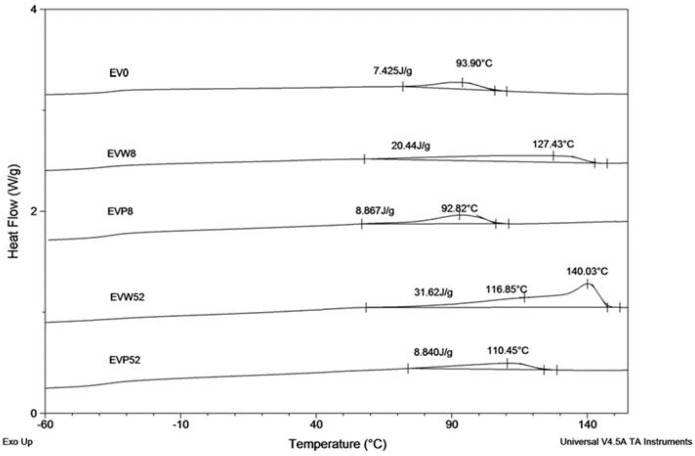
The DSC curves (cooling run) of EV samples before (EV0) and after 8 and 52 weeks of incubation in water (W) and paraffin (P).

The results of the DSC analysis, described below, confirm the observed behavior. Figure 6 shows the DSC curves (during cooling at 10 °C/min from melt) of EV samples after the specified incubation times in the cosmetic media.

Samples incubated in water showed changes in the crystallization region due to the fact that after degradation of the PLA component, the remaining sample contained only the PBAT component (Figure 6 shows DSC curves of samples EVW8 and EVW52, respectively after 8 and 52 weeks of incubation). For the sample EVW8 after 8 weeks of incubation in water, the broad region with crystallization temperature (*T*_c_) at 127.4 °C was observed. The DSC curve of the EVW52 sample showed a broad double exotherm in the crystallization region. The crystallization pick at 140 °C was noticed in the EV sample after 52 weeks of degradation in water. The observed value of the crystallization temperature was characteristic for the PBAT with the increased content of aromatic segments [27]. The increased content of the PBAT aromatic segments, due to privileged hydrolytic degradation of aliphatic structural units, was confirmed also by the NMR results (see Figure 5) [17]. Comparing the DSC curves of EVP8 and EVP52 samples incubated in paraffin with the initial DSC curve of EV0, it was found that the PBAT and PLA blend in paraffin did not show differences within the crystallization region nor the incubation time. 

Changes in the thermal properties during the degradation process are summarized in Table 3.

The DSC data (from the heating run) for samples after 8 (EVW8) and 52 (EVW52) weeks of incubation confirmed that after incubation in water, due to complete degradation of the PLA component, the sample remaining contained only the PBAT part. The melting temperature (*T_m_*_PLA_) and glass transition temperature (*T_g_*_PLA_) of the PLA component were not observed in the DSC experiment. This lack of the PLA component in studied samples (EVW8 and EVW52) was also confirmed by ^1^H NMR and ESI-MS analysis. Contradictorily, no changes in the thermal properties for the samples incubated in paraffin after 8 and 52 weeks (EVP8 and EVP52) were observed.

**Figure 7:**
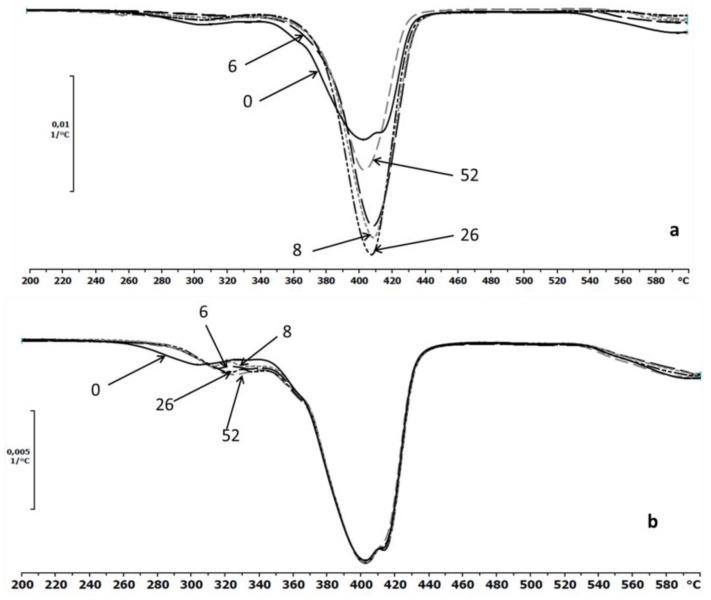
The DTG curves of EV samples before (0) and after 6, 8, 26 and 52 weeks of incubation in water (**a**) and paraffin (**b**).

The thermal properties of the EV samples were also determined by TGA. The first derivative TG and DTG curves were the best indicator of the temperature at which the various stages of the thermal decomposition took place [4]. The DTG thermograms for the investigated samples before and after the specified incubation times in the degradation media (water and paraffin) were presented in Figure 7a,b, respectively. After degradation in water, minor differences of thermal stabilities of the samples were observed, while no changes were observed for samples incubated in paraffin (Figure 7b).

The thermograms of the samples after degradation in paraffin indicated a lack of changes in the maximum temperature decomposition peaks that was characteristic for the aliphatic–aromatic copolyester. The slight differences are only shown on the part of the curve that likely corresponded to the thermal decomposition of the PLA. 

**Figure 8:**
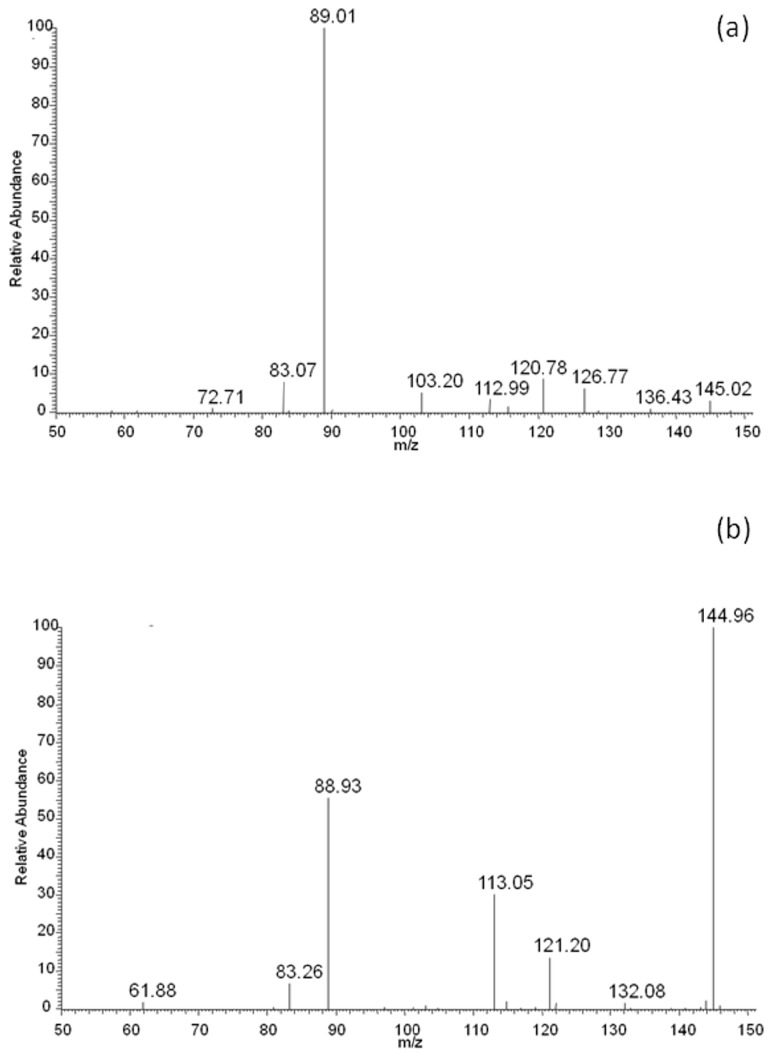
ESI-MS spectra (in negative-ion mode) in the range of *m/z* 50–150 of degradation products released to the water after 2 (**a**) and 52 (**b**) weeks of hydrolytic degradation of the PBAT and PLA blend at 70 °C.

The above presented results indicated that lactic and adipic acids, as well as oligomers, should have formed as degradation products of the EV blend during its incubation in water, and should have been present in the degradation medium. For confirmation of this, the ESI-MS analysis was performed. The rate of hydrolytic degradation of PLA was higher than the rate of hydrolytic degradation of PBAT. Therefore, during degradation, the lactic acid was observed in the water medium (*m/z* 89; Figure 8a) after 2 weeks of degradation, while with the progress of degradation, the intensity of the signal corresponding to adipic acid (*m/z* 145; derivative from butylene adipate unit of PBAT) in the ESI-MS spectrum was observed, and gradually increased to finally outgrow the intensity of the signal corresponding to lactic acid (Figure 8b). 

It is interesting to notice that no signals related to PLA oligomers were observed in the water medium. The fast hydrolytic degradation rate of PLA in the blend as evidenced by NMR and the lack of PLA oligomers in the degradation medium (as evidenced by ESI-MS analysis during incubation in water), may have indicated a scission mechanism on the chain-end [28]. Generally, polyesters undergo hydrolysis via random-chain scission. The chain-end scission mechanism could have resulted from the short distance between the carbonyl and the alkoxy groups of the PLA closed in the polymer matrix, and/or increased acidic conditions that created a good environment for the occurrence of chain-end hydrolysis in a privileged way [29].

**Figure 9:**
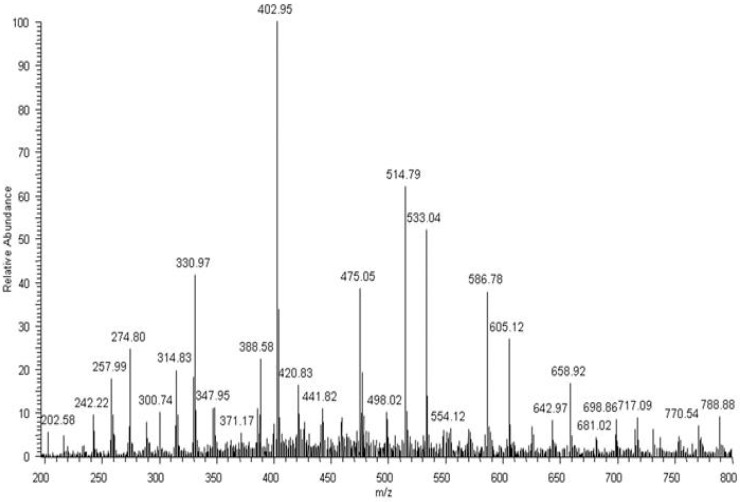
ESI-MS spectrum (in positive-ion mode) of degradation products released to the water after 52 weeks of hydrolytic degradation of the PBAT and PLA blend at 70 °C.

After 52 weeks of incubation in water, degradation products of PBAT were detected in the medium. The PBAT copolymer contained butylene terephtalate (BT; 220 Da) and butylene adipate (BA; 200 Da) units. Moreover, different combinations of building blocks derived from butanediol (B; 72 Da), terephthalic acid (T; 148 Da) and adipic acid (A; 128 Da) could occur in the degradation products, due to the fact that ester bonds undergo random scission during hydrolysis. As a result, there were three possible combinations of end groups: (i) two hydroxy groups, in the case where building blocks derived from butanediol were at both ends of the molecule; (ii) two carboxy groups, in the case where building blocks derived from terephthalic acid and/or adipic acid were at both ends of the molecule; and (iii) one hydroxy end group and one carboxy end group. Considering the possible combination of building blocks and various end groups, the structures were assigned for the ions visible in ESI-MS spectrum (Figure 9): for example, the ion at *m/z* 533 was assigned to the structure H(BT)(BA)BOH + Na^+^, which contains two hydroxy end groups; the ion at *m/z* 587 was assigned to the structure HA(BT)_2_OH + H^+^, which contains two carboxy end groups; and the ion at *m/z* 659 was assigned to the structure H(BT)_2_(BA)OH + H^+^, which contains one hydroxy end group and one carboxy end group. Proposed structures were compatible with previously published results [12]. Thus, the ESI-MS technique enables the monitoring of degradation products of both components of the blend, i.e., PBAT and PLA released to water during hydrolytic degradation. In the early stage of hydrolytic degradation, mostly the lactic acid was observed. However, within the degradation progress, degradation products of PBAT were easily detected in the water medium.

### 3.2. (Bio)degradation Test under Industrial Composting Conditions

The above results indicate that the PBAT and PLA blend possessed a good stability during the aging in cosmetic media (paraffin), and it can be assumed that final products made from this blend could be inert enough in contact with the cosmetics containing oily ingredients. Moreover, the sachet made from the blend of PBAT and PLA should be stable during a longer term of cosmetic storage, compared to packages made only from PLA [2,3]. Furthermore, such new packaging materials considered as compostable should be subjected to organic recycling after use [30]. For this reason, the EV and sachet samples were tested under industrial composting conditions in the Biodegma system.

**Figure 10:**
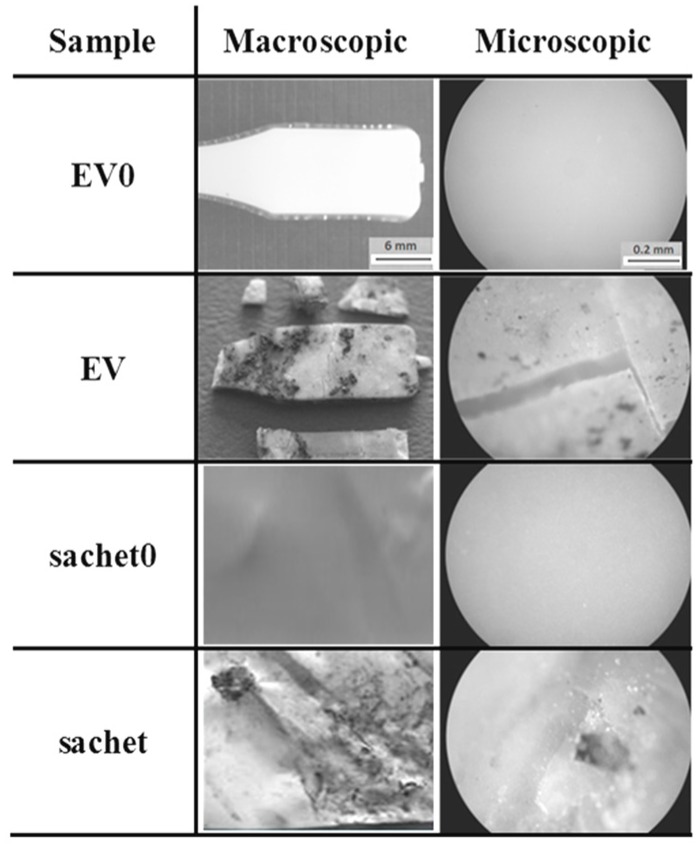
Changes of the studied samples’ surfaces after 6 weeks of the industrial composting process.

The studied samples observed under the optical microscope (magnification 100×) before incubation had a smooth surface. After the composting process, the macro- and microscopic changes of the EV sample and sachet surfaces were observed, and are presented in Figure 10.

After 6 weeks of incubation on the EV surface, scratches and cracks appeared, while on the sachet’s surfaces, the pits were clearly visible. Additionally, the microscopic examination indicated that the damage process of the EV samples occurred layer-by-layer, while for the sachet, on whole surface the holes were formed. The same effect for the thicker EV sample incubated in water was observed after 8 weeks of the aging test (comparison: Figure 2 and Figure 3).

**Figure 11:**
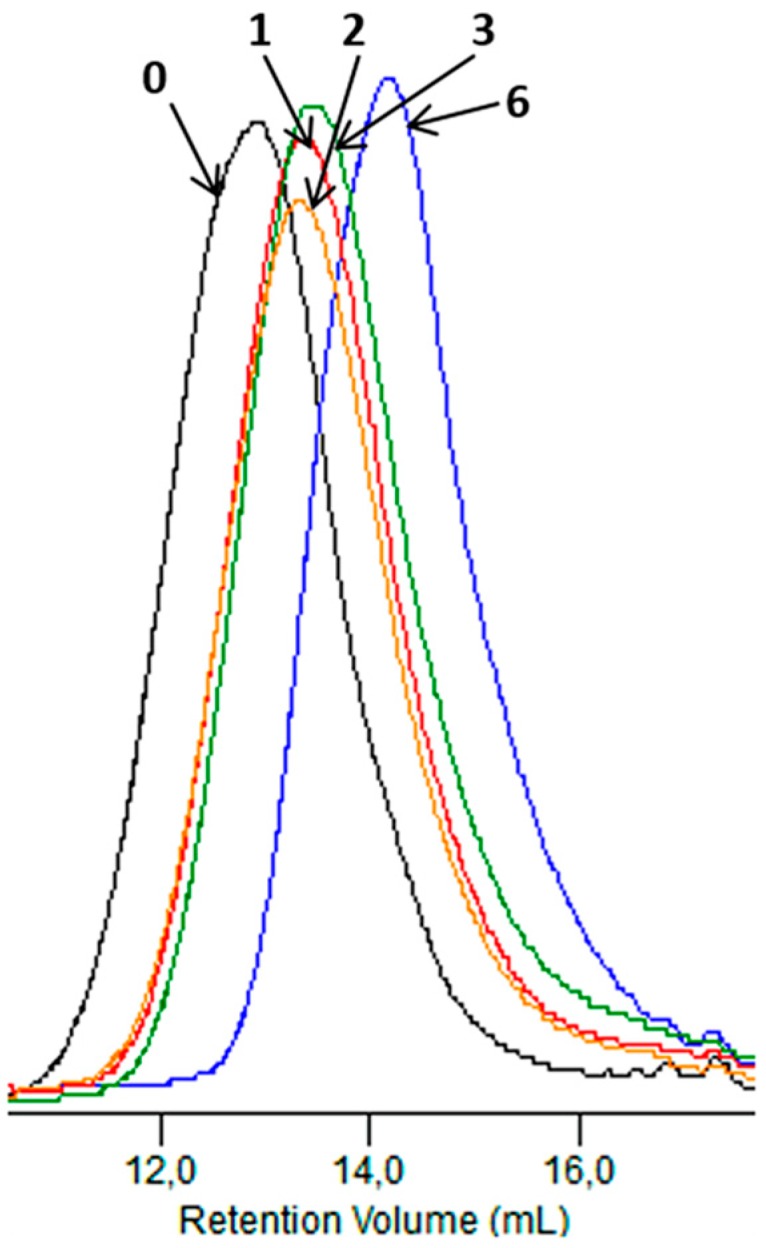
GPC elugrams of the sachet before (0) and after 1, 2, 3 and 6 weeks of the industrial composting process.

Figure 11 shows GPC elugrams depicting changes in the molar mass of the sachet after specified incubation times in the Biodegma system. During the composting process, the systematic decreasing of the molar mass of samples studied was noticed. This may have indicated that the main process occurring during the composting test under the industrial condition was hydrolytic degradation [21].

**Figure 12:**
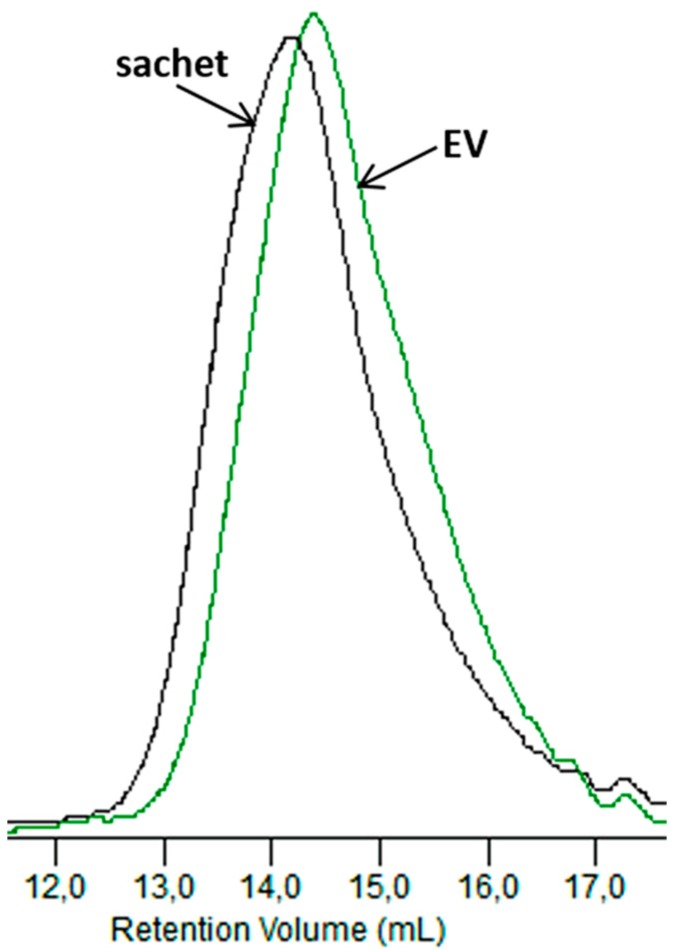
GPC elugrams of the sachet and EV samples after 6 weeks of the industrial composting process.

Figure 12 compares the GPC elugrams for the dumbbell-shaped sample and the sachet after 6 weeks of incubation in the Biodegma system. 

A more significant shift to a low molar mass was observed for the thicker sample of EV. Therefore, the products’ shapes and especially their thicknesses could influence processes occurring during composting under the industrial condition. A similar impact of the thickness on the degradation profile was observed during the aging test under laboratory conditions, and could be explained as the result of the autocatalytic effect [31].

The results of the current study do not reflect the changes of the blend of PBAT with 12 mol % of PLA during storage. However, according to the Ecoflex Brochure, the results of the aging test of films made of a composition of PBAT with PLA in a standard climate (23 °C; 50% relative atmospheric humidity) showed that mechanical properties of film change in the course of 2 years of storage time [32]. Therefore, the storage time may affect the stability of the final packaging product from the PBAT and PLA blend. However, our results showed a good stability of this blend during degradation in cosmetic media (paraffin), and this material may be recommended for long-shelf-life compostable packaging of cosmetics, especially those with oily ingredients.

In conclusion, the integrated FEAPM approach presented in this prediction study revealed that the blend of PBAT (containing 47 mol % of aromatic segments) with 12 mol % of PLA may be a promising candidate for the manufacturing of compostable packages of cosmetics, especially those with oily ingredients. Additionally, the results evidenced by the NMR and ESI-MS analyses indicated chain-end scission during the incubation in water of the PLA component of the blend studied.

## Figures and Tables

**Table 1:** Thermal properties of materials studied.

Sample	*T_g_*_PBAT_ [°C]	Δ*c_p_*_PBAT_ [J/g °C]	*T_m_*_PBAT_ [°C]	Δ*H_m_*_PBAT_ [J/g]	*T_g_*_PLA_ [°C]	Δ*c_p_*_PLA_ [J/g °C]	*T_m_*_PLA_ [°C]	Δ*H_m_*_PLA_ [J/g]
EV	−29.7/49.1	0.19/0.22	125.5	7.1	60.4	0.07	156.2	0.2
Sachet	−28.3/47.9	0.18/0.18	121.1	9.6	62.1	0.08	152.7	0.2

*T_g_*_PBAT_ and *T_g_*_PLA_—glass transition temperatures; *T_m_*_PBAT_ and *T_m_*_PLA_—melting temperatures; Δ*H_m_*_PBAT_ and Δ*H_m_*_PLA_—melting enthalpies; and ΔC*_p_*_PBAT_ and ΔC*_p_*_PLA_—heat capacity increments of PBAT and PLA components of studied samples, respectively.

**Table 2:** The mechanical properties of the sachet.

Stress at Break	24.6 MPa
Elongation at break	754.7%
Tear resistance	7.73 N
Shrinkage	47.3%
Coefficient of friction	0.2
Welding	0.3 s (115 °C)

**Table 3:** Thermal properties of samples during incubation in water (W) and paraffin (P).

Sample	*T_gPBAT_* [°C]	Δ*c_p_*_PBAT_ [J/g °C]	*T_m_*_PBAT_ [°C]	Δ*H_m_*_PBAT_ [J/g]	*T_g_*_PLA_ [°C]	Δ*c_p_*_PLA_ [J/g °C]	*T_m_*_PLA_ [°C]	Δ*H_m_*_PLA_ [J/g]
EV0	−29.7/49.1	0.19/0.22	125.5	7.1	60.4	0.07	156.2	0.2
EVW8	−35.5/36.5	0.29/0.29	141.1	14.5	-	-	-	-
EVW52	−35.0/50.4	0.21/0.33	154.9	24.6	-	-	-	-
EVP8	−30.8/45.3	0.16/0.14	126.7	5.6	60.7	0.07	152.2	0.5
EVP52	−35.1/43.3	0.22/0.25	129.1	5.4	53.4	0.04	153.6	0.7

*T_g_*_PBAT_ and *T_g_*_PLA_—glass transition temperatures; *T_m_*_PBAT_ *and T_m_*_PLA_—melting temperatures; Δ*H_m_*_PBAT_ and Δ*H_m_*_PLA_—melting enthalpies; and ΔC*_p_*_PBAT_ and ΔC*_p_*_PLA_—heat capacity increments of PBAT and PLA component of studied samples, respectively.

## References

[1] Capra P., Briasco B., Sorrenti M., Catenacci L., Sachet M., Perugini P. (2014). Preliminary evaluation of packaging-content interactions: Mechanical and physicochemical characterization of polylactide bottles. J. Appl. Polym. Sci..

[2] Rydz J., Adamus G., Wolna-Stypka K., Marcinkowski A., Misiurska-Marczak M., Kowalczuk M.M. (2013). Degradation of polylactide in paraffin and selected protic media. Polym. Degrad. Stab..

[3] Rydz J., Wolna-Stypka K., Musioł M., Szeluga U., Janeczek H., Kowalczuk M. (2013). Further evidence of polylactide degradation in paraffin and in selected protic media. A thermal analysis of eroded polylactide films. Polym. Degrad. Stab..

[4] Rydz J., Wolna-Stypka K., Adamus G., Janeczek H., Musioł M., Sobota M., Marcinkowski A., Krzan A., Kowalczuk M. (2015). Forensic engineering of advanced polymeric materials. Part 1—Degradation studies of polylactide blends with atactic poly[(R,S)-3-hydroxybutyrate] in paraffin. Chem. Biochem. Eng. Q..

[5] Sikorska W., Adamus G., Dobrzynski P., Libera M., Rychter P., Krucinska I., Komisarczyk A., Cristea M., Kowalczuk M. (2014). Forensic engineering of advanced polymeric materials—Part II: The effect of the solvent-free non-woven fabrics formation method on the release rate of lactic and glycolic acids from the tin-free poly(lactide–*co*–glycolide) nonwovens. Polym. Degrad. Stab..

[6] Musioł M., Sikorska W., Adamus G., Kowalczuk M. (2016). Forensic engineering of advanced polymeric materials. Part III—Biodegradation of thermoformed rigid PLA packaging under industrial composting conditions. Waste Manag..

[7] Musioł M., Sikorska W., Adamus G., Janeczek H., Kowalczuk M., Rydz J. (2016). (Bio)degradable polymers as a potential material for food packaging: Studies on the (bio)degradation process of PLA/(R,S)–PHB rigid foils under industrial composting conditions. Eur. Food Res. Technol..

[8] Musioł M., Rydz J., Janeczek H., Radecka I., Jiang G., Kowalczuk M. (2017). Forensic engineering of advanced polymeric materials Part IV: Case study of oxo-degradable polyethene commercial bags—Aging in biotic and abiotic environment. Waste Manag..

[9] Rychter P., Biczak R., Herman B., Zawierucha I., Musioł M., Sobota M., Kowalczuk M. (2011). Environmental degradation of aromatic-aliphatic polyester blends. Evaluation of degradation products in soil and their phytotoxicological impact. Pol. J. Environ. Stud..

[10] Vroman I., Tighzert L. (2009). Biodegradable Polymers. Materials.

[11] Siegenthaler K., Kunkel A., Skupin G., Yamamoto M. (2012). Ecoflex^®^ and Ecovio^®^: Biodegradable, performance-enabling plastics. Adv. Polym. Sci..

[12] Song J., Šišková A., Simons M.G., Kowalski W.J., Kowalczuk M.M., van den Brink O.F. (2011). LC-Multistage Mass Spectrometry for the characterization of poly(butylene adipate–*co*–butylene terephthalate) copolyester. J. Am. Soc. Mass Spectrom..

[13] Rychter P., Kawalec M., Sobota M., Kurcok P., Kowalczuk M. (2010). Study of aliphatic-aromatic copolyester degradation in sandy soil and its ecotoxicological impact. Biomacromolecules.

[14] Georgiopoulos P., Kontou E., Niaounakis M. (2014). Thermomechanical properties and rheological behavior of biodegradable composites. Polym. Compos..

[15] Kontou E., Spathis G., Georgiopoulos P. (2014). Modeling of nonlinear viscoelasticity–viscoplasticity of bio-based polymer composites. Polym. Degrad. Stab..

[16] Georgiopoulos P., Kontou E. (2015). The effect of wood-fiber type on the thermomechanical performance of a biodegradable polymer matrix. J. Appl. Polym. Sci..

[17] Gan Z., Kuwabara K., Yamamoto M., Abe H., Doi Y. (2004). Solid-state structures and thermal properties of aliphatic-aromatic poly(butylene adipate–*co*–butylene terephthalate) copolyesters. Polym. Degrad. Stab..

[18] Tachibana Y., Maeda T., Ito O., Maeda Y., Kunioka M. (2009). Utilization of a biodegradable mulch sheet produced from poly(lactic acid)/Ecoflex^®^/modified starch in mandarin orange groves. Int. J. Mol. Sci..

[19] International Organization for Standardization (2012). EN ISO 527-2: Plastics—Determination of Tensile Properties—Part 2: Test Conditions for Moulding and Extrusion Plastics.

[20] International Organization for Standardization (1999). ISO 15814: Implants for Surgery—Copolymers and Blends Based on Polylactide—In Vitro Degradation Testing.

[21] Musioł M.T., Rydz J., Sikorska W.J., Rychter P.R., Kowalczuk M.M. (2011). A preliminary study of the degradation of selected commercial packaging materials in compost and aqueous environments. Pol. J. Chem. Technol..

[22] ASTM (2014). ASTM E 1356: Standard Test Method for Assignment of the Glass Transition Temperatures by Differential Scanning Calorimetry.

[23] Andersson S.-R., Hakkarainen M., Albertsson A.-C. (2010). Tuning the polylactide hydrolysis rate by plasticizer architecture and hydrophilicity without introducing new migrants. Biomacromolecules.

[24] Cam D., Suong-Hyu H., Ikada Y. (1995). Degradation of high molecular weight poly(lactide) in alkaline medium. Biomaterials.

[25] Zbik M., Horn R.G., Shaw N. (2006). AFM study of paraffin wax surfaces. Colloids Surf. A.

[26] Wu T.M., Hsu S.F., Shih Y.F., Liao C.S. (2008). Thermal degradation kinetics of biodegradable poly(3-hydroxybutyrate)/layered double hydroxide nanocomposites. J. Polym. Sci. A.

[27] Ahn B.D., Kim S.H., Kim Y.H., Yang J.S. (2001). Synthesis and characterization of the biodegradable copolymers from succinic acid and adipic acid with 1,4-butanediol. J. Appl. Polym. Sci..

[28] Gleadall A., Pan J., Kruft M.-A., Kellomäki M. (2014). Degradation mechanisms of bioresorbable polyesters. Part 1. Effects of random scission, end scission and autocatalysis. Acta Biomater..

[29] Shih C. (1995). Chain-end scission in acid catalyzed hydrolysis of poly(d,l-lactide) in solution. J. Control. Release.

[30] Brostow W., Hagg Lobland H.E. (2017). Materials. Introduction and Applications.

[31] Espartero J.L., Rashkov I., Li S.M., Manolova N., Vert M. (1996). NMR Analysis of low molecular weight poly(lactic acid)s. Macromolecules.

[32] Brochure KT/KC, E 100, 2009. Biodegradable Polymers—Inspired by Nature Ecoflex^®^, Ecovio^®^. http://www.plasticsmanufacturers.co.za/basf_pdf/Ecoflex_Brochure.pdf.

